# ASO Author Reflections: Surgical Radicality Versus Aggressive Biology: Small Bowel and Ileocecal Involvement Predict Poorer Survival Despite Complete Cytoreduction in FIGO IIIC–IV Tubo-Ovarian and Primary Peritoneal Carcinoma

**DOI:** 10.1245/s10434-026-19529-w

**Published:** 2026-04-27

**Authors:** Ingo B. Runnebaum, Angela Kather

**Affiliations:** 1 Department of Gynecology and Reproductive Medicine, Jena University Hospital, Friedrich- Schiller-University Jena, Jena, Germany; 2 RU21 GmbH, Jena, Germany; 3 Zentrum für Alternsforschung Jena - Aging Research Center Jena, Jena, Germany

## Past

Advanced tubo-ovarian and primary peritoneal carcinoma has long reinforced a surgical reflex: if complete macroscopic resection is achievable, pursue it. Yet, even after technically flawless, maximum-effort surgery, a subset of patients recurs early, raising the persistent question of whether specific anatomic sites convey biologic risk beyond global surgical complexity and tumor burden.

## Present

In a uniform maximum-effort cohort of 302 all-comer patients (FIGO IIIC–IV) with a high complete macroscopic resection rate of 85%, small bowel involvement and, independently, ileocecal involvement remained significantly associated with inferior survival despite complete cytoreduction.^1^ These findings reframe such patterns: they are not merely determinants of surgical feasibility, but rather high-risk anatomic features that persist even after a tumor-free endpoint is achieved. Consequently, we need to place greater emphasis on the preoperative identification of extensive small bowel and ileocecal disease including selective open diagnostic laparoscopy when imaging is inconclusive to refine multidisciplinary conference (MDC) counseling regarding treatment sequencing and expected oncological benefit, thereby providing greater prognostic certainty for both clinicians and patients.^2,3^

## Future

Recent data from the TRUST trial presented at ASCO 2025 demonstrate improved progression-free survival with primary vs. interval debulking in audited high-volume centers, although without a statistically significant overall survival advantage or quality-of-life differences between arms.^4^ Together with our findings, this supports prospective studies that explicitly capture small bowel and ileocecal involvement preoperatively and evaluate sequencing strategies in this subgroup, while integrating translational work to define potential molecular correlates including distinct tumor immune profiles. In the interim, when extensive small bowel or ileocecal involvement is identified and upfront morbidity risk is high, neoadjuvant chemotherapy with planned interval debulking may be a leading option in selected patients within a shared decision-making framework. When complete macroscopic resection is achievable without small bowel or ileocecal resection, upfront maximum-effort cytoreduction remains the mainstay of care.

*Medicus curat* *,* *natura sanat.*
